# Adverse outcome of infants with metastatic neuroblastoma, MYCN amplification and/or bone lesions: results of the French Society of Pediatric Oncology

**DOI:** 10.1054/bjoc.2000.1412

**Published:** 2000-10

**Authors:** V Minard, O Hartmann, M C Peyroulet, J Michon, C Coze, A S Defachelle, O Lejars, Y Perel, C Bergeron, P Boutard, G Leverger, J L Stephan, A Thyss, P Chastagner, G Couillault, C Devalck, P Lutz, F Mechinaud, F Millot, D Plantaz, X Rialland, H Rubie

**Affiliations:** On behalf of the Société Française d’Oncologie Pédiatrique;, Department of Pediatrics, Institut Gustave Roussy, 39 rue Camille Desmoulins, Villejuif Cedex, 94805, France

**Keywords:** neuroblastoma, infants, metastasis, MYCN, bone lesions

## Abstract

To assess the relevance of MYCN amplification and bone lesions in stage 4 neuroblastoma (NB) in infants aged <1 year, 51 infants with stage 4 NB were enrolled. Three groups of patients were defined according to the type of metastases and the resectability of the primary tumour. Group I comprised 21 infants with radiologically detectable bone lesions, Group II 22 patients with an unresectable primary tumour and Group III eight patients with only metaiodobenzylguanidine (MIBG) skeletal uptake. MYCN oncogene content was assayed in 47/51 tumours and found to be amplified in 17 (37%). The 5-year event-free survival (EFS) rate of these 51 infants was 64.1% (± 7.1%). In a univariate analysis, bone lesions, MYCN amplification, urinary vanillylmandelic/homovanillic acid ratio and serum ferritin levels adversely influenced outcome. In the multivariate analysis, radiologically detectable bone lesions were the most powerful unfavourable prognostic indicator: the EFS rate was 27.2% for these infants compared to 90% for infants without bone lesions (*P* < 0.0001). Our data emphasize the poor prognosis of infants affected by stage 4 NB with bone lesions, especially when associated with MYCN amplification. Given the poor results in this group whatever the treatment, new therapeutic approaches need to be investigated in the future. © 2000 Cancer Research Campaign


*British Journal of Cancer* (2000) **83**(8), 973–979
© 2000 Cancer Research Campaign
doi: 10.1054/ bjoc.2000.1412, available online at http://www.idealibrary.com on IDEAL®

### Adverse outcome of infants with metastatic neuroblastoma, MYCN amplification and/or bone lesions: results of the French Society of Pediatric Oncology

**V Minard, O Hartmann[1], MC Peyroulet, J Michon, C Coze, AS Defachelle, O Lejars, Y Perel, C Bergeron, P Boutard, G Leverger, JL Stephan, A Thyss, P Chastagner, G Couillault, C Devalck, P Lutz, F Mechinaud, F Millot, D Plantaz, X Rialland and H Rubie**

On behalf of the Société Française d'Oncologie Pédiatrique; [1]Department of Pediatrics, Institut Gustave Roussy, 39 rue Camille Desmoulins, 94805 Villejuif Cedex, France

**Summary** To assess the relevance of MYCN amplification and bone lesions in stage 4 neuroblastoma (NB) in infants aged <1 year, 51 infants with stage 4 NB were enrolled. Three groups of patients were defined according to the type of metastases and the resectability of the primary tumour. Group I comprised 21 infants with radiologically detectable bone lesions, Group II 22 patients with an unresectable primary tumour and Group III eight patients with only metaiodobenzylguanidine (MIBG) skeletal uptake. MYCN oncogene content was assayed in 47/51 tumours and found to be amplified in 17 (37%). The 5-year event-free survival (EFS) rate of these 51 infants was 64.1% (± 7.1%). In a univariate analysis, bone lesions, MYCN amplification, urinary vanillylmandelic/homovanillic acid ratio and serum ferritin levels adversely influenced outcome. In the multivariate analysis, radiologically detectable bone lesions were the most powerful unfavourable prognostic indicator: the EFS rate was 27.2% for these infants compared to 90% for infants without bone lesions ($P < 0.0001$). Our data emphasize the poor prognosis of infants affected by stage 4 NB with bone lesions, especially when associated with MYCN amplification. Given the poor results in this group whatever the treatment, new therapeutic approaches need to be investigated in the future. © 2000 Cancer Research Campaign

**Keywords**: neuroblastoma; infants; metastasis; MYCN; bone lesions

Neuroblastoma (NB) is one of the most common solid tumours usually affecting children in the first years of life. Approximately 30% of patients are under 1 year at the time of diagnosis (Bernstein et al, 1992). In infants, the most frequent stages are stage 1 (35%) followed by stage 4-s (20%) and stage 4 (14%) (Castel Sanchez et al, 1997). When Evans et al (1971) proposed a staging system for neuroblastoma, they gave a clear definition of stage 4-s disease. Essentially observed in infants under 1 year-of-age, this special stage was defined as disease that would otherwise be stage 1 or 2, but with dissemination confined to one or more of the following sites: liver, skin or bone marrow (without radiological evidence of bone metastases at complete skeletal survey). The revised International Neuroblastoma Staging System (Brodeur et al, 1993) stipulated an upper limit at 10% of bone marrow involvement for stage 4-s disease. Thus, infants with stage 4 disease are patients with metastatic disease which does not correspond to the 4-s definition.

Recently described genetic markers are being analysed extensively in children over 1 year with stage 4 neuroblastoma, but their prognostic significance remains unclear.

The overriding objectives of the present study were to undertake an analysis of prognostic factors in an unselected cohort of infants with stage 4 neuroblastoma, and especially to assess the prognostic significance of MYCN amplification (MNA) and bone lesions in this population.

In 1990, a national prospective study (NBL 90) was initiated that was to include all consecutive infants with metastatic NB diagnosed in the member institutions of the French Society of Pediatric Oncology (SFOP). We report herein the results of the SFOP-NBL 90 study.

*Received 27 March 2000*
*Revised 9 June 2000*
*Accepted 27 June 2000*

*Correspondence to*: O Hartmann

### PATIENTS AND METHODS

### Patient population

Between February 1990 and February 98, 21 member institutions of the SFOP participated in this study. All infants under 1 year-of-age with newly diagnosed stage 4 neuroblastoma were eligible for entry. Previous chemotherapy was an exclusion criterion, but primary surgery was not. Data concerning all infants were reviewed to verify their consistency with the revised International Neuroblastoma Staging System (INSS) and Response Criteria (INRC) for diagnosis, staging and assessment of response (Brodeur et al, 1993). A total of 55 infants were enrolled in the study. Four were ineligible, due to uncertainty regarding compliance and follow-up evaluation ($n = 3$), or to insufficient available data ($n = 1$). The present analysis therefore concerns 51 infants. It reports the outcome of infants as of November 1998, 9 months after inclusion of the last case.

The diagnosis of neuroblastoma was based on conclusive clinical, laboratory and imaging findings and was always confirmed by histology and/or cytology. The primary tumour was evaluated by computed tomography scan or magnetic resonance imaging (MRI) and/or ultrasonography and iodine$^{123}$ or $^{131}$metaiodobenzylguanidine (MIBG) scintigraphy (McEwan et al, 1985). The complete metastatic work-up included a skeletal study by MIBG (or a Technetium$^{99m}$ scan in the absence of MIBG-uptake at the site of the primary tumour), a complete examination of the skeleton by X-rays and extensive bone-marrow staging (according to INSS criteria). When MIBG-uptake was detected in metastases, several views were obtained to confirm that uptake was exclusively localized in the skeleton and not in soft tissues. In addition, the films and scans were centrally reviewed if MIBG scan showed uptake in one or several skeletal sites. Urinary vanillylmandelic (VMA) and homovanillic (HVA) acid levels and dopamine excretion according to creatinine concentration (Bertani-Dziedzic et al, 1990) were measured, as were serum neuron-specific enolase (NSE) (Zeltza et al, 1986), ferritin (Hahn et al, 1980), and lactate dehydrogenase (LDH) (Shuster et al, 1992) levels.

Analysis of the MYCN oncogene was planned for all tumours. Results were considered reliable only if tumour material exhibited over 10% of neuroblasts. MYCN genomic content was determined by Southern or slot blot using MYCN second exon probes. MYCN amplification (MNA) was defined as ≥ 10 copies per haploid genome. Other biological investigations such as evaluation of the DNA index (Look et al, 1984) or a search for deletion of the short arm of chromosome 1 (Caron, 1993) were optional.

Labreveux de Cervens et al (1994) defined three groups of infants with stage 4 neuroblastoma. These groups appeared different in terms of median age, response to chemotherapy and long-term survival. In our study, the 51 infants with stage 4 disease were then divided into these three groups:

*Group I* comprised 21 infants whose bone lesions were detected by X-ray. None of these patients had a normal skeletal MIBG scan (or Tc$^{99m}$ scan); they all exhibited skeletal uptake.

*Group II* comprised 22 infants with metastatic disease but with no detectable bone lesion neither by MIBG nor by X-ray. The primary tumour in this group was considered unresectable and associated with metastases, especially in the liver, skin, or slight bone-marrow involvement. Tumours were defined as unresectable when they traversed and invaded the midline structures, usually those encasing large vessels, or when their size, structure, or location prohibited surgery without a high risk of rupture or major surgical complications. The latter included some large thoracic tumours that compressed the upper respiratory tract without necessarily crossing the midline extensively. All dumbell tumours, with or without neurologic impairment, were deemed unresectable (Rubie et al, 1997).

*Group III* consisted of eight infants, in which bone lesions were not detected by X-ray but the MIBG scan showed uptake in one or several skeletal sites. This group includes patients with MIBG features consistent with cortical bone disease and those with features consistent with bone marrow involvement.

Thus, 51 evaluable infants with stage 4 disease entered the study, 21 had radiologically detectable bone lesions and 30 had not (22 unresectable primary tumours and eight only exhibiting MIBG skeletal uptake).

### Therapeutic protocol

### *Chemotherapy*

*Group I* Chemotherapy consisted of four courses of cyclophosphamide–doxorubicin–vincristine CAdO (Coze et al, 1997), followed by surgery if complete remission was obtained at all metastatic sites and high-dose chemotherapy (HDC) with stem cell transplantation (SCT) in case of MNA. In the event of no response or progressive disease after the first two courses of CAdO, treatment was switched for etoposide and platinum compounds, as in the case of patients with stage 4 neuroblastoma over 1 year-of-age (Coze et al, 1997), followed by surgery and HDC with SCT if complete remission (CR) or partial remission (PR) was obtained.

*Group II* These patients received the same treatment as that administered for localized unresectable neuroblastoma. Chemotherapy consisted of two courses of carboplatin and etoposide (Frappaz et al, 1992), followed by two courses of CAdO. All patients received four courses of chemotherapy, and radical surgery was then attempted. After surgery, chemotherapy was indicated for residual disease and/or lymph-node involvement (one course of each combination).

*Group III* Treatment of these patients was left to the discretion of the physician. Patients were treated either like stage 4-s (Michon et al, 1993) or like stage 4 without HDC if MYCN was not amplified (MNN). In keeping with the NBL 90 protocol, stage 4-s lesions were treated according to the evolutionary course of the disease. Initial treatment included six courses of cyclophosphamide and vincristine (CO), followed by surgery. If no response was obtained or disease progressed after CO, second-line therapy using irradiation or chemotherapy was given, followed by surgical excision of the primary tumour.

According to the NBL90 protocol, all patients with metastatic neuroblastoma (groups I, II and III) and MNA received high-dose chemotherapy followed by SCT.

### *Radiotherapy*

After November 1992, because of a high incidence of local relapses, locoregional irradiation was recommended for infants whose tumour exhibited MNA, regardless of age or the quality of the surgical excision. A total dose of 25 Gy was delivered in daily fractions of 1.5–1.8 Gy each.

### Evaluation of reponse to therapy

Response to therapy was assessed according to INSS criteria (Brodeur et al, 1971). Response was defined as follows: complete remission (CR) = no detectable disease; very good partial remission (VGPR) = only a small local tumour residue (< 10% of the initial size) but no detectable disease at any site of metastasis; partial remission (PR) ≥ 50% reduction of all measurable and evaluable disease. All other situations were considered as failures (mixed response (MR), no response (NR) and progressive disease (PD)). Tumour response was evaluated during and at the end of

 © 2000 Cancer Research Campaign induction therapy, 1 month after surgery and 6–8 weeks following HDC, and at least every 3 months thereafter.

### Statistical analysis

A comparison of infants whose tumours were MYCN amplified (MNA) and those whose tumours were not MYCN amplified (MNN) was performed for each variable with the $\chi^2$ test corrected for heterogeneity or Fisher's exact test (Peto et al, 1977). The probabilities of survival were calculated from the time-of-diagnosis to relapse, or death, or last follow-up, according to the Kaplan–Meir product-limit method (Fleiss, 1981). In the event-free-survival (EFS) analysis, disease progression or relapse, and death, whatever the reasons, were considered as events. Multivariate assessment of EFS was performed using Cox's proportional hazards model and curves were compared using the log-rank test (Cox, 1977).

### RESULTS

### Patient characteristics

Patient characteristics are listed in Table 1. The median age was 7 months (range: newborn–12 months). The primary tumour was abdominal in 37 patients (73%). Ten children had a dumbbell tumour (seven thoracic, one cervical, one abdominal, and one pelvic tumour). Forty-five of 51 patients with metastatic disease had multiple sites of metastasis. The bone marrow ($n$ = 36) and the liver ($n$ = 25) were the most frequently involved. MYCN oncogene content was assayed in 47 tumours and found to be amplified (≥ 10 copies) in 17 (37%).

**Table 1** Patient characteristics

| Characteristic | *n* | % |
|---|---|---|
| Patients | 51 | 100 |
| Sex | | |
| Male | 29 | 57 |
| Female | 22 | 43 |
| Age | | |
| Median ((min–max) months) | 7 (0–12) | |
| Mean ((standard deviation) months) | 6.3 (3.7) | |
| < 6 months | 23 | 45 |
| Site of primary tumour | | |
| Abdomen | 37 | 72 |
| Thorax | 7 | 14 |
| Others | 7 | 14 |
| Dumbell | 10 | 20 |
| Size of primary tumour | | |
| T1: < 5 cm | 7 | 14 |
| T2: 5–10 cm | 34 | 66 |
| T3: > 10 cm | 8 | 16 |
| Multiple | 2 | 4 |
| Group | | |
| Group I (bone lesions) | 21 | 41 |
| Group II (unresectable primary tumour) | 22 | 43 |
| Group III (MIBG skeletal uptake) | 8 | 16 |
| Metastases | | |
| Bone | 21 | 41 |
| Bone marrow | 36 | 70 |
| Liver | 25 | 49 |
| Subcutaneous nodules | 10 | 20 |
| Intraparenchymal CNS | 2 | 4 |
| Others | 3 | 6 |
| Elevated NSE (> 2N) ($n$ = 37 measured) | 31 | 84 |
| Elevated LDH (> 2N) ($n$ = 38 measured) | 14 | 37 |
| Elevated Ferritin (> 2N) ($n$ = 36 measured) | 6 | 17 |
| Elevated urinary catecholamine excretion HVA/VMA > 1 | 26 | 51 |
| MYCN oncogene analysis (47 measured) > 10 copies | 17 | 36 |

### Neuroblastoma stage 4 with bone lesions (group I)

The bone metastases of the 21 patients in this group were detected by both X-ray and MIBG or $Tc^{99m}$ scan. Patient characteristics are listed in Table 2. The sex ratio was 16 males/5 females, and the median age was 9 months (range = 1–12 months). The primary tumour was abdominal in 20 patients. All these 21 patients had a single or multiple bone lesions on X-ray films. Three of them presented a single site of uptake on MIBG scan (and one or several radiologically detectable bone lesions), whereas 17 had multiple sites of osteomedullary uptake. One last patient had a negative MIBG scan even at the primary tumour site, but the $Tc^{99m}$ bone scan showed a single positive lesion. MYCN oncogene content was found to be amplified (≥ 10 copies) in 13/21 (62%).

Seven of these 21 infants achieved a CR of all metastatic sites following first-line chemotherapy. Four of them were consolidated with high-dose chemotherapy (HDC) because of MYCN amplification. Of these four, two are alive in first CR with a follow-up of 69 and 9 months post-diagnosis, one died of infection 28 months after HDC, and one relapsed 7 months after HDC and died. One other patient with MNA, who was not consolidated, relapsed 2 months after surgery and died. The two patients with MNN who achieved a CR were treated with conventional chemotherapy and surgery. One of them is alive in first CR having attained a follow-up of 75 months, and the other died of treatment-related complication after HDC performed in second PR.

The fate of the other 14 patients was as follows: in one, disease progressed rapidly during induction and he died. One infant died of infection after two courses of CAdO. The twelve remaining infants achieved a partial response (PR) of their metastases after first-line chemotherapy (CAdO). Two of them (MNA) died of disease progression, and ten achieved a CR or VGPR on second-line therapy (etoposide/platinum). Nine of these ten were further consolidated with HDC. Four of them are alive and free of disease (> 64, 49, 22 and 12 months) (three MNN) and the other five died of disease. The remaining patient (MNN), who entered VGPR after second-line therapy, was not consolidated with HDC and is alive and free of disease (> 65 months).

Hence 8/21 patients with bone lesions are alive and free of disease, of whom three had MNA and five have MNN tumour.

### Neuroblastoma stage 4 without bone lesion (group II and III)

#### *Group II*

Bone metastases were not detectable either by X-ray or MIBG scan in the 22 infants in this group (Table 2). The sex ratio was 8 males/14 females, and the median age was 4 months (range = 0–11 months). The primary tumour was abdominal in 12 patients, thoracic in five, cervical in two, cervico-thoracic in two and pelvic in one. A dumbbell tumour was found in seven patients. MYCN oncogene content was assayed in 18/22 tumours and found to be amplified in four (22%).

Ten infants without MYCN amplification were treated according to the NBL90 protocol. All are alive in first CR with a

© 2000 Cancer Research Campaign

**Table 2** Patient characteristics according to bone lesions

| Factor | Stage IV with bone lesions | Stage 4 without bone lesion | *P* |
|---|---|---|---|
| Cases (*n*) | 21 | 30 | |
| Sex Male/Female | 16/21 | 13/30 | 0.02 |
| Age | | | |
| median (months (min–max)) | 9 (0–12) | 4 (0–11) | < 0.0001 |
| < 6 months | 3/21 | 20/30 | |
| Site of primary tumour = abdomen | 20/21 | 17/30 | 0.01 |
| Metastases | | | |
| Bone marrow | 18 | 18 | 0.047 |
| Liver | 6 | 19 | 0.01 |
| Subcutaneous nodules | 3 | 7 | 0.49 |
| Intraparenchymal CNS | 2 | 0 | 0.16 |
| Others | 2 | 1 | 0.56 |
| Elevated NSE (> 2N (*n* = 37 measured) | 14/18 | 17/19 | > 0.99 |
| Elevated Ferritin (> 2N) (*n* = 36 measured) | 4/15 | 2/21 | 0.09 |
| Elevated LDH (> 2N) (*n* = 38 measured) | 8/19 | 6/19 | 0.08 |
| Urinary catecholamine excretion | | | |
| HVA/VMA < 1 | 16/21 | 10/30 | 0.002 |
| MYCN oncogene analysis (47 measured) | | | |
| > 10 copies | 13/21 | 4/26 | 0.001 |

follow-up of 5–68 months post-diagnosis. Four other infants, with an MNN tumour, were treated like stage 4-s disease (protocol violations). Only one achieved a CR and is alive and free of disease (> 25 months). Disease progressed on therapy in the other three and they entered CR after second-line therapy and are alive (> 82, 25 and 6 months). Of the four other infants with MNA tumour, two were consolidated and are alive and free of disease (> 51 and 44 months). The third was treated only with the conventional NBL90 chemotherapy and he is alive in first CR (> 53 months). The fourth patient died of a surgical complication during initial biopsy.

The MYCN status was unknown (MNU) in four patients because the tumour-cell content of the sample was poor (*n* = 2), or because it was not analysed (*n* = 2). One of them died of sepsis after four courses of chemotherapy. Another, treated according to the protocol, is alive in first CR with a follow-up of 65 months. The remaining two patients were treated like stage 4-s disease and are alive and free of disease (> 69 and 33 months).

In summary, in this group, 20/22 infants are alive in CR or PR, of whom 14 had an MNN tumour, three an MNA tumour and three were MNU.

### *Group III*

Skeletal MIBG uptake was evidenced in eight infants, but their X-ray films were normal. The sex ratio was 5 males/3 females, and the median age was 5 months (range = 0–11 months). The primary tumour was retroperitoneal in five patients. Two patients presented a single site of uptake on the skeletal MIBG scan, whereas six patients had multiple sites of osteomedullary uptake. No patient had an MNA tumour.

Four patients were treated according to the protocol used for stage 4 disease without HDC and are alive and free of disease in first CR (> 91, 71, 40 and 31 months). The four others received the same treatment administered for stage 4-s disease: one died of progression despite a second-line therapy and three are alive (> 85, 42 and 17 months), two of whom have remained in PR despite combination chemotherapy, radiotherapy and surgery.

Seven of eight infants are therefore alive in CR or PR in this group.

### Prognostic factors

The factors likely to influence event-free survival (EFS) were first studied by univariate analysis (logrank test). Factors which were significant or of borderline significance were further analysed by multivariate analysis. The projected overall survival and EFS rates at 5 years are 67.2% (± 6.1%) and 64.1% (± 7.1%) for all 51 infants with stage 4 neuroblastoma. The results of the univariate analysis are given in Table 3. The comparison of EFS between patients with or without MNA indicated significantly poorer survival in the subgroup with an MNA tumour (*P* = 0.0004) (Figure 1). However, the most powerful prognostic indicator was bone lesions (Figure 2): indeed EFS was far better in the 30 infants without a bone lesion compared to the 21 with bone lesions (90% (± 5.5%) vs 27.2% (± 10.6%), *P* < 0.0001). Regarding tumour markers, only the serum ferritin level and VMA/HVA ratio appeared to affect outcome, particularly when ferritin was over twice the normal value (*P* = 0.008). Conversely, sex, age-at-diagnosis, the site of the primary tumour, as well as histologically proven lymph-node involvement, had no impact on outcome. Multivariate analysis was performed using the Cox regression method with stratification on age, size and the site of the primary tumour, MNA and bone lesions. The only significant prognostic factor was bone lesions (*P* = 0.001), with a relative risk of 12.06 (95% CI, 2.71–53.5). The EFS rate at 5 years is 11.7% (± 10.6%) for the MNA subgroup with bone lesions and 75% (± 21.7%) for the MNA subgroup without bone lesions. This difference is not statistically significant (*P* = 0.10) due to the small number of infants.

### DISCUSSION

To our knowledge, this is the first multicentre prospective study to focus on infants with metastatic neuroblastoma (NB) with the aim of evaluating both the clinical relevance of MYCN amplification and of bone lesions in such a population. Two groups were defined based on radiological findings, the first comprising infants with bone lesions detected by X-ray (group I) and the second those with no radiologically detected bone lesion (groups II and III).

 *© 2000 Cancer Research Campaign*

**Table 3** Prognostic factors: univariate analysis

| Factor | *n* patients (*n* events) | *P* (log-rank) |
|---|---|---|
| Sex | | |
| Male | 29 (10) | 0.99 |
| Female | 22 (7) | |
| Age | | |
| < 6 months | 23 (5) | 0.16 |
| > 6 months | 28 (12) | |
| Site of primary tumour | | |
| Abdominal | 37 (15) | 0.14 |
| Nonabdominal | 14 (2) | |
| Bone lesions | | |
| Yes | 21 (14) | < 0.0001 |
| No | 30 (3) | |
| Size of primary tumour | | |
| T1 | 7 (2) | NE |
| T2 | 34 (11) | |
| T3 | 8 (4) | |
| multiple | 2 (0) | |
| Urinary cathecholamines | | |
| HVA/VMA > 1 | 26 (13) | 0.008 |
| HVA/VMA < 1 | 25 (4) | |
| NSE (*n* = 37) | | |
| Normal | 6 (0) | NE |
| Abnormal > 2N | 31 (9) | |
| Ferritin (*n* = 36) | | |
| Normal | 30 (6) | 0.0008 |
| Abnormal > 2N | 6 (5) | |
| LDH (*n* = 38) | | |
| Normal | 24 (5) | 0.17 |
| Abnormal > 2N | 14 (6) | |
| NMA (*n* = 47) | | |
| < 10 copies | 30 (5) | 0.0004 |
| > 10 copies | 17 (11) | |

NE = not evaluable

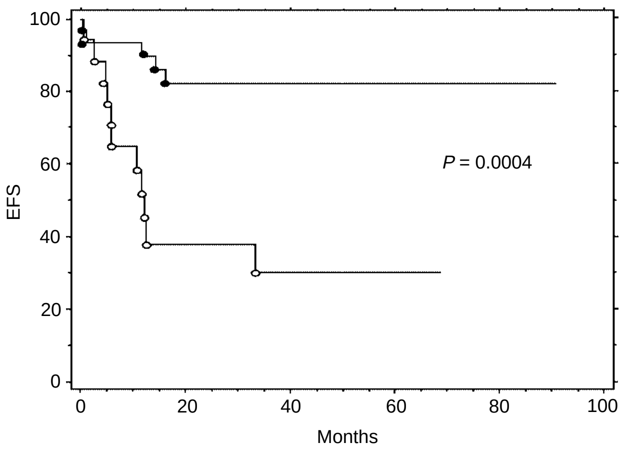

**Figure 1** Event-free survival according to MYCN status; ● MYCN non-amplified; ○ MYCN amplified

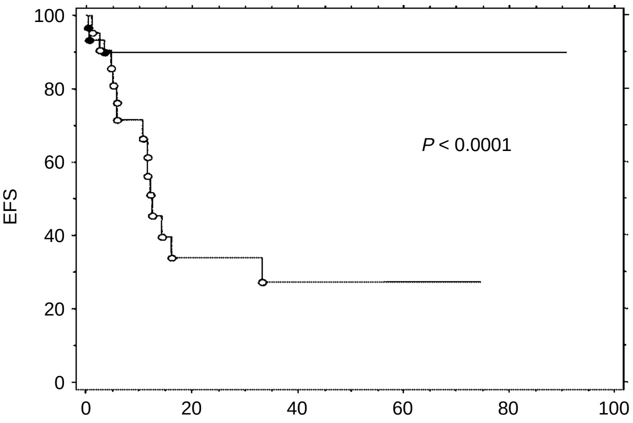

**Figure 2** Evert-free survival according to bone lesions; ● No bone lesion; ○ Bone lesions

Our patient characteristics were comparable to those of other published studies (Paul et al, 1991; Labreveux de Cervens et al, 1994; Strother et al, 1995; Lampert et al, 1997). The classic prognostic factors analysed in NB to date were the focus of this study. In terms of age at diagnosis, other authors have already underscored the existence of a more favourable prognosis for disease in younger infants (0–6 months) (De Bernardi et al, 1992; Labreveux de Cervens et al, 1994). De Bernardi et al (1992) published that infants who were 6–11 months old with stage 4 disease had the worst prognosis. In a smaller series, De Cervens et al (1994) further substantiated this conclusion, but in their study stage 4 patients were not only the oldest but also had bone lesions. Our data confirm that infants with bone lesions are indeed the oldest, as were those with MNA tumours, however age in itself had no impact whatsoever on outcome in the univariate analysis.

With respect to sex as prognostic factor, some investigators have suggested that it has an effect on survival conferring a more favourable outcome on females (Carlsen et al, 1985). An excess of male patients in our study did in fact have stage 4 NB and bone lesions but this did not lead to a difference in survival between genders; male and female patients had similar survival rates in the univariate analysis.

The site as a prognostic factor has also been purported to impact on survival. Goon et al (1984) found that neuroblastoma confined to the thorax have the most favourable outcome. In our study, the primary sites were not statistically different between the three groups and had no effect on outcome.

Evidence for the prognostic significance of MYCN oncogene amplification is inconsistent and controversial. MYCN was found to be correlated with a poor prognosis in neuroblastoma (Seeger et al, 1985; Nakagawara et al, 1987; Look et al, 1991; Brodeur et al, 1992) and was demonstrated to be the most relevant adverse prognostic indicator in localized NB (Rubie et al, 1997). The legitimacy of this finding however has remained open to debate in patients with stage 4 disease > 1 year-of-age. MYCN amplification has also been singled out as conferring a poor prognosis (Bowman et al, 1997; Dubois et al, 1999) in patients with stage 4 NB under 1

year, but this finding was not corroborated by others (Lampert et al, 1997). Indeed, Lampert et al (1997) reported no difference in the probability of survival between infants with stage 4 NB, with or without a MYCN amplified tumour. In their recent study, Dubois et al (1999) showed that MYCN amplification correlated with increased frequency of bone and intracranial or orbital metastases. In our study, MYCN amplification as a univariate factor correlated significantly with survival ($P$ = 0.0004) (Figure 1) and MNA and bone lesions were also significantly correlated ($P$ = 0.001) (Table 2).

Although certain other biological prognostic indicators, such as serum ferritin and urinary catecholamine excretion (HVA/VMA ratio) appeared to be significant in the univariate analysis, they were finally of marginal importance in the multivariate analysis. This was related to the fact that the VMA/HVA ratio was significantly correlated with bone lesions and MNA, and that there were major variations between serum ferritin determinations between the different centres.

Event-free survival rate at 5 years is 64.1% (± 7.1%) for our entire study population. Paul et al (1991) reported a similar 5-year EFS (75%) for 24 patients and, given the favourable outcome of this series of infants with metastatic NB < 1 year-of-age, did not advocate using HDC in these patients. Strother et al (1995) reported a 5-year actuarial survival rate of 60% for 58 patients. Although survival rates appear comparable, these authors did not analyse the prognostic value of bone lesions, which was the strongest prognostic indicator in the multivariate analysis in our study. Based on our findings, we consider that patients < 1 year with stage 4 NB with bone lesions should be distinguished from patients with the same disease stage and age but no bone lesions, because their outcome is totally different and, as a consequence, the former require more aggressive treatment. Evans et al (1971) already demonstrated that overt bone lesions in infants, which were to be clearly distinguished from bone-marrow involvement without positive radiological findings, virtually always portended a fatal outcome. Since then, the difference between stage 4-s and 4 disease has been clearly established. In addition, MIBG scintigraphy has made it possible to identify a specific group of infants whose X-rays are normal but in whom osteomedullary MIBG uptake is evidenced (group III in this study). Our data suggest that disease in this specific group differs from that of stage 4 NB with radiologically detectable bone lesions because the long-term outcome appears better than that of patients with radiologically detectable bone lesions. Moreover, the prognosis appears to be different from that of stage 4-s disease. In our opinion, the prognosis in group III is similar to that of stage 4-s when bone marrow involvement exceeds the 10% limit stipulated for classification as 4-s by the INSS (Brodeur et al, 1993). We consider skeletal MIBG uptake to be related to greater bone-marrow involvement, which would account for its role as a prognostic factor. Like stage 4 NB, this stage with MIBG uptake alone requires more aggressive conventional chemotherapy to achieve a complete remission.

With respect to infants affected by a stage 4 unresectable primary tumour (group II), our study confirms the excellent outcome of this stage at this age (< 1 year) (Labreveux de Cervens et al, 1994). Indeed, only toxicity is incriminated in the 2/22 deaths in group II. The treatment and the prognosis of this stage are the same as that of stage 3 neuroblastoma in patients under 1 year-of-age at diagnosis (Rubie et al, 1997). According to the three groups, three different treatment regimens were used for this study in which prognostic factors were analysed. Hence, stage 4 neuroblastoma with bone lesions were treated with the most aggressive therapy. Despite the fact that treatment is an important part of outcome, in this series patients with bone lesions had the worst prognosis.

Finally, this study has shown that radiologically detectable bone lesions are a strong prognostic factor conferring a very poor prognosis on infants with bone lesions, especially when their tumours are MNA. Given the poor results of this group, whatever the treatment, new therapeutic approaches need to be investigated in the future.

### ACKNOWLEDGEMENTS

We thank Lorna Saint Ange for revising the manuscript.

#### REFERENCES

Bernstein ML, Leclerc JM, Bunin G, Brisson L, Robinson L, Shuster J, Byrne T, Gregory D, Hill G, Dougherty G, Scriver C, Lemieux B, Tuchman M and Woods WG (1992) A population-based study of neuroblastoma incidence, survival, and mortality in North America. *J Clin Oncol* **10**: 323–329

Bertani-Dziedzic L, Dziedzic SW and Gitlow SE (1990) Catecholamine metabolism in neuroblastoma, in Pochelldly C (ed): Neuroblastoma: Tumor Biology and therapy. Boca Raton, FL, CRC; pp 69–91

Bowman LC, Castleberry RP, Cantor A, Joshi V, Cohn SL, Smith EI, Yu A, Brodeur GM, Hayes FA and Look AT (1997) Genetic staging of unresectable or metastatic neuroblastoma in infants: a Pediatric Oncology Group Study. *J Natl Cancer Inst* **89**: 373–380

Brodeur GM, Pritchard J, Berthold F, Carlsen NLT, Castel V, Castleberry RP, De Bernardi B, Evans AE, Favrot M, Hedborg F, Kaneko M, Kemshead J, Lampert F, Lee REJ, Look T, Pearson ADJ, Philip T, Roald B, Sawada T, Seeger RC, Tsuchida Y and Voute PA (1993) Revisions of the International Criteria for Neuroblastoma Diagnosis, Staging and response to Treatment. *J Clin Oncol* **11**: 1466–1477

Brodeur GM, Azar C, Brother M, Hiemstra J, Kaufman B, Marshall H, Moley J, Nakagawara A, Saylors R, Scavarda N, Schneider S, Wasson J, White P, Seeger R, Look T and Castleberry RP (1992) Neuroblastoma: Effects of genetics on prognosis and treatment. *Cancer* **70**: 1685–1694

Caron H (1993) In neuroblastoma alletic loss chromosome 1 p and the presence of additional chromosome 17 q material are both associated with an unfavorable outcome. Twenty-fifth Annual Meeting of the International Society of Pediatric Oncology, San Francisco, CA, October 5–9

Castel Sanchez V, Melero Moreno C, Garcia-Miguel Garcia-Rosados A, Navajas Gutierrez A, Ruiz Jimenez JI, Navarro Fos S, Garin Valle JC and Galbe Sada M (1997) Neuroblastoma en ninos menores de 1 ano. *An Esp Pediatr* **47**: 584–590

Cox DR (1977) The analysis of Binary Data. London, England, Chapman & Hall

Coze C, Hartmann O, Michon J, Frappaz D, Dusol F, Rubie H, Plouvier E, Leverger G, Bordigoni P, Behar C, Beck D, Mechinaud F, Bergeron C, Plantaz D, Otten J, Zucker JM, Philip T and Bernard JL (1997) NB 87 induction protocol for stage 4 neuroblastoma in children over 1 year of age: A report from the French Society of Pediatric Oncology. *J Clin Oncol* **15**: 3433–3440

De Bernardi B, Pianca C, Boni L, Brisigotti M, Carli M, Bagnulo S, Corciulo P, Mancini A, De Laurentis C, Di Tullio MT, Cordero Di Montezemolo L, Lanino E, Clerico A, Rogers DW and Bruzzi P (1992) Disseminated Neuroblastoma (Stage IV and IV-S) in the first year of life. *Cancer* **70**: 1625–1633

Carlsen NL, Schroeder H, Bro PV, Erichsen G, Hamborg-Pedersen B, Jensen KB and Nielsen OH (1985) Neuroblatomas treated at the four major child oncologic clinics in Denmark 1943–1980: an evaluation of 180 cases. *Med Pediatr Oncol* **13**: 180–186

Dubois G, Kalika Y, Lukens J, Brodeur GM, Seeger RC, Atkinson JB, Haase GM, Black CT, Perez C, Shimada H, Gerbing R, Stram DO and Matthay KK (1999) Metastatic sites in stage IV and IVS neuroblastoma correlate with age, tumor biology, and survival. *J Pediatr Hematol-Oncol* **21(3)**: 181–189

Evans AE, D'Angio GJ and Randolph J (1971) A proposed staging for children with neuroblastoma. *Cancer* **27**: 374–378

Fleiss JI (1981) Statistical Methods for Rates and Proportions (ed 2). New York, NY, Wiley

Frappaz D, Michon J, Hartmann O, Bouffet E, Lejars O, Gentet JC, Chastagner P, Sariban E, Brugiere L, Zucker JM, Lemerle J and Philip T (1992) Etoposide

 *© 2000 Cancer Research Campaign*

and carboplatin in neuroblastoma: A French Society of Pediatric Oncology phase II study. *J Clin Oncol* **10**: 1592–1601

Goon HK, Cohen DH and Harvey JG (1984) Review of thoracic neuroblastoma. *Aust Paediatr J* **20**: 17–21

Hahn HWL, Levy HM, Evans AE (1980) Serum ferritin as a guide to therapy in neuroblastoma. *Cancer Res* **40**: 1411–1413

Labreveux de Cervens C, Hartmann O, Bonnin F, Couanet D, Valteau-Couanet D, Lumbroso J, Behar C, Martelli H and Lemerle J (1994) What is the prognostic value of osteomedullary uptake on MIBG Scan in neuroblastoma patients under one year of age? *Med Pediatr Oncol* **22**: 107–114

Lampert F, Chritiansen H, Terpe HJ and Berthold F (1997) Disseminated neuroblastomas under 1 year of age: cell biology and prognosis. *Jour of neuro-Onc* **31**: 181–184

Look AT, Hayes FA, Nitschke R, McWilliams NB and Green AA (1984) Cellular DNA content as a predictor of response to chemotherapy in infants with unresectable neuroblastoma. *N Engl J Med* **311**: 231–235

Look AT, Hayes FA, Shuster JJ, Douglass EC, Castleberry RP, Bowman LC, Smith EI and Brodeur GM (1991) Clinical relevance of tumor cell ploidy and N-*Myc* gene amplification in childhood neuroblastoma: A Pediatric Oncology Group Study. *J Clin Oncol* **9**: 581–591

McEwan AJ, Shapiro B, Sisson JC, Beierwaltes WH and Ackery DM (1985) Radio-iodobenzylguanidine for the scintigraphic location and therapy of adrenergic tumors. *Semin Nucl Med* **25**: 132–153

Michon J, Hartmann O, Frappaz D, Chastagner P, Rubie H, Perel Y, Sariban E, Wyss M, Legall E, Courbon B, Philip T, Lemerle J and Zucker JM (1993) Adaptation of the treatment to clinical presentation in stage 4s neuroblastoma. Results of the French Society of Pediatric Oncology NBL 90 prospective study. *Med Ped Oncol* **21**: 585 (Abstract p 122)

Nakagawara A, Ikeda K, Tsuda T and Hisashi K (1987) N-*Myc* oncogene amplification and prognostic factors of neuroblastoma in children. *J Pediatr Surg* **22**: 895–898

Paul SR, Tarbell NJ, Korf B, Kretschmar CS, Lavally B and Grier H (1991) Stage 4 neuroblastoma in infants. *Cancer* **67**: 1493–7

Peto R, Pike MC, Armitage P, Breslow NE, Cox DR, Howard SV, Mantel N, McPherson K, Peto J and Smith PG (1977) Design and analysis of clinical trials requiring prolonged observation of each patient. *Br J Cancer* **35**: 1–39

Rubie H, Hartmann O, Michon J, Frappaz D, Coze C, Chastagner P, Baranzelli MC, Plantaz D, Avet-Loiseau H, Bernard J, Delattre O, Favrot M, Peyroulet MC, Thyss A, Perel Y, Bergeron C, Courbon-Collet B, Vannier JP, Lemerle J and Sommelet D (1997) N-*Myc* Gene amplification is a major prognostic factor in localized neuroblastoma: Results of the French NBL 90 Study. *J Clin Oncol* **15**: 1171–1182

Seeger RC, Brodeur GM, Sather H, Dalton A, Siegel SE, Wong KY and Hammond D (1985) Association of multiples copies of the N- *Myc* oncogene with rapid progression of neuroblastoma. *N Engl J Med* **313**: 1111–1116

Shuster JH, McWilliams NB, Castelberry R, Nitschke R, Smith EI, Altshuler G, Kun L, Brodeur G, Joshi V, Vietti T and Hayes FA (1992) Serum lactate deshydrogenase in childhood neuroblastoma. *Am J Clin Oncol* **15**: 295–303

Strother D, Shuster JJ, McWilliams N, Nitschke R, Smith EI, Joshi V, Kun L, Hayes FA and Castleberry R (1995) Results of pediatric Oncology Group Protocol 8104 for infants with stage D and DS Neuroblastoma. *Journ of Pediatr Hem-Oncol* **17(3)**: 254–259

Zeltza PM, Marangos PJ, Evans AE and Schneider SL (1986) Serum neuron specific enolase in children with neuroblastoma. Relationship to stage and disease course. *Cancer* **57**: 1230–1234

*© 2000 Cancer Research Campaign* 
